# Deciding to lead: a qualitative study of women leaders in emergency medicine

**DOI:** 10.1186/s12245-018-0206-7

**Published:** 2018-11-16

**Authors:** Mindi Guptill, Ellen T. Reibling, Kathleen Clem

**Affiliations:** 1 Department of Emergency Medicine, Loma Linda University School of Medicine, 11234 Anderson St., MC A108, Loma Linda, CA 92354 USA; 2 University of Central Florida College of Medicine, Orlando, FL USA

**Keywords:** Emergency medicine, Women, Leadership, Career progression, Gender diversity

## Abstract

**Background:**

The aim of this study is to highlight career paths of senior women leaders in academic emergency medicine (EM) to encourage younger women to pursue leadership.

**Methods:**

This was a qualitative study using semi-structured interviews with female EM leaders. We interviewed 22 recognized female leaders selected using criterion-based sampling and a standardized script of open-ended questions derived from the Intelligent Career Model. Questions were related to job purpose, skills, and networking. Interviews were transcribed verbatim and three trained reviewers analyzed transcripts following grounded theory principles and using Dedoose®. Researchers used an iterative process over several meetings to produce the final set of codes and themes.

**Results:**

Our iterative process identified four themes: women leaders made an intentional decision to pursue opportunities to influence emergency medicine, women sought out natural mentors and sponsors to facilitate career development, women leaders intentionally planned their out of work life to support their leadership role, and an important focus for their work was to help others achieve excellence.

**Conclusions:**

Our study provides insights from senior female leaders in EM; supporting the value of women pursuing leadership. There is a widely acknowledged need to diversify leadership and support gender-specific needs to develop women leaders in medicine. Becoming a woman leader in EM means making intentional decisions and taking risks. Leaders found benefits in natural mentors and sponsors. Those relationships have power to change the trajectory of emerging women leaders by identifying and reinforcing potential. Work/life balance remains an area which requires intentional planning. Woman leaders encourage succession planning and corroborate the need for increasing the percentage of women leaders to benefit the organizational culture. Leadership in academic medicine is changing with reorientation of a largely autocratic, vertically oriented hierarchy into a more democratic, consensus-driven, and horizontally organized management structure which should complement the strengths women bring to the leadership table.

## Introduction

Women are underrepresented in emergency medicine (EM) leadership despite greater numbers of women entering medical schools (Table 1) [1]. The highest proportion of female leaders is in Obstetrics and Gynecology (22%), lowest is Orthopedic Surgery (0%), with EM in the middle at 10% [2]. While the percentage of women choosing EM has increased 11.5% since 2001 (28.3% to 39.8%), the number of female EM chairs in 2018 was only 11 out of more than 90 departments [1].

**Table 1:** Academic leadership by gender

	Men *n* (%)	Women *n* (%)	Total
Division heads and academic chairs [1]	2331 (84)	437 (16)	2768
Emergency medicine chair [1]	86 (89)	11 (11)	97
Emergency medicine faculty [1]	2867 (66)	1484 (34)	4351

The impetus for increasing women in leadership includes stronger corporate social and ethical behavior (i.e., less fraud), more innovation, and in many cases increased revenue [3]. More women leaders have the potential to influence equality at lower levels and decreases incidents of harassment because the presence of more women “makes such behavior unacceptable.” ([3, 4] p. 4) Qualitative studies with women directors suggest that organizations appoint at least three women: “one is token, two is a presence, and three is a voice,” which translates to an aspirational goal of 30% for most boards [5]. Translated to medicine, more women in leadership can improve health care delivery resulting in better patient care at a lower cost.

The barriers to increasing the ranks of women are not completely clear. Earlier studies in EM emphasized career advancement as a key predictor of career satisfaction [6]. Women, however, are less “apt to see themselves as qualified for top positions” despite equivalent training and credentials (p. 1057) [7]. Women are less likely to achieve senior positions or to remain in academia [8, 9]. Females leaving academia reported few role models who combined careers with rearing children and lack of collaborative work cultures [7, 10–12]. Women need role models who combine technical and emotional support with help building professional networks [10]. There is a need for a “new paradigm” to explain the fact that increasing numbers of women in academic medicine are not resulting in more women at senior-level positions [13].

Previous qualitative studies highlighted the experience of women who left academia but did not describe the experience of women who stayed in leadership roles [12, 14]. We felt that a qualitative study of high achieving women leaders would provide a deeper understanding of their sustained careers in EM. We wanted to understand the ways in which female leaders viewed their career progression in order to support emerging leaders choosing a similar path.

## Methods

### Study design and setting

**Fig. 1:**
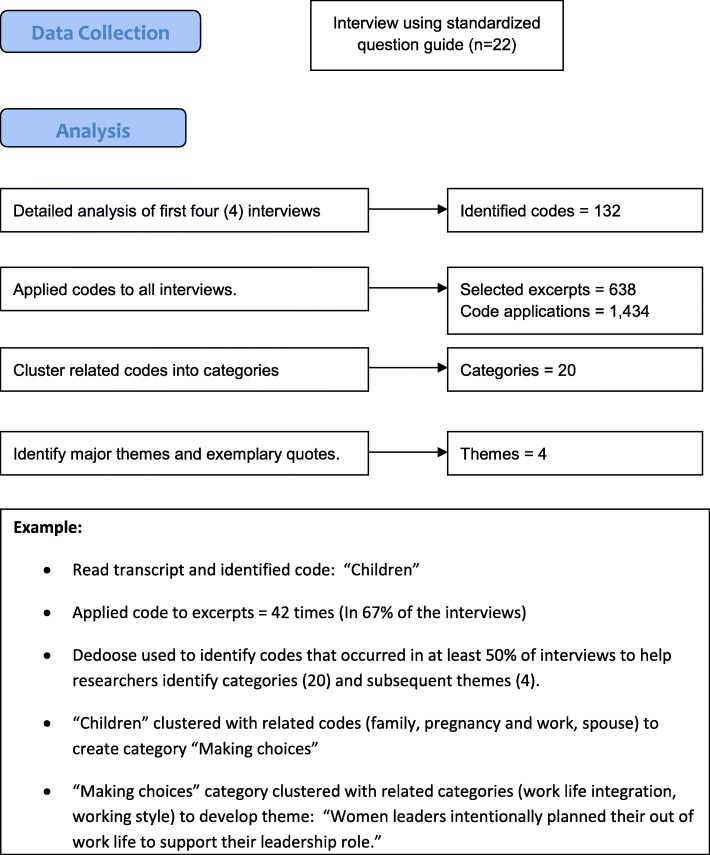
Methodology flowchart

The purpose of this study was to explore the leadership pathway among women in EM. We used phenomenological qualitative methods including semi-structured interviews and open-ended questions to encourage reflective discussions from the respondents [15]. We conducted interviews of EM female leaders over 8 months. Following the Consolidated criteria for Reporting Qualitative Research (COREQ) checklist [16], we continued interviews until theoretical saturation was achieved whereby our concepts were well-developed, diverse perspectives were represented in the sample, and further interviews were not likely to yield significant new insights [17]. Published studies employed similar methods [12, 13]. The study was approved for a waiver of written consent by the Loma Linda University Institutional Review Board (#5130197). Participants provided verbal consent, including permission to audio record the interviews and publish excerpts with identifying information removed (Fig. 1).

### Participant selection

We used purposive sampling to compile a list of female academic EM department chairs and previous female presidents of the American College of Emergency Medicine. Participants were recruited via email and telephone. We collected limited demographic data to protect anonymity of participants.

### Data collection

#### Semi-structured interview

An initial set of interview questions was developed using the intelligent career model (ICM) that sets career development in a social context through growth in three domains: knowing why (focus on purpose), how (focus on skill building), and whom (focus on networking) [18]. The interview guide was piloted with a qualitative research expert, and the study principal investigator (KC) reviewed the guide with American Association of Women Emergency Physicians (AAWEP) leadership, the study sponsor. Questions were revised and iteratively modified after interviews as themes emerged that merited further explanation. A PhD researcher trained in qualitative approaches conducted the interviews, which were transcribed verbatim with identities removed, and stored on a password-protected computer.

### Outcomes and primary data analysis

We employed several strategies to ensure trustworthiness in qualitative research, including a standardized interview guide of open-ended questions to bolster diverse and rich responses. We minimized assumptions by reviewing the first four interviews before completing the final codebook and maintained an audit trail so that the study could be replicated [19].

Transcripts were uploaded into Dedoose® (Version 6.0.19, Los Angeles, 2014) for coding and analysis. Three research team members independently analyzed interviews by coding initial transcripts and identifying additional codes. The first four interviews were used to edit the coding scheme to develop themes that clustered similar responses. Codes were routinely reevaluated to ensure consistency and to identify codes needing clarification. At least two independent team members (MG, ER, KC) applied the final coding scheme to the remaining transcripts and discrepancies were resolved by discussion. After all transcripts were analyzed, authors met four times to summarize codes into major themes and subthemes. The study team collaborated on a final thematic framework and identified exemplary quotes relevant to each theme.

## Results

### Subject characteristics

We completed 22 interviews lasting an average of 65 min. All participants with exception of one were or had been an EM chair for at least 8 years and worked in EM a mean of 27 years; 47% were currently serving in that role. All of the subjects held medical degrees and 40% held additional graduate degrees in nursing, business, or public health.

### Main results

Our iterative process resulted in a final codebook of four major themes: women leaders made an intentional decision to pursue opportunities to influence emergency medicine, women sought out natural mentors and sponsors to facilitate career development, women leaders intentionally planned their out of work life to support their leadership role, and an important focus for their work was to help others achieve excellence.

Selected quotes are displayed in Table 2. Quotes in the text are italicized.

**Table 2:** Clustered themes and illustrative quotations from interviews with senior female leaders in EM

Themes	Illustrative quotations
Women leaders made an intentional decision to pursue opportunities to influence emergency medicine.	
Made strategic decisions and took risks	I think people in emergency medicine, we are not very good at saying, “You need me to do that?” I mean, we are really bad at walking past a crisis and not responding to it. So, I guess I learned first of all, transparency—being fair and being transparent are about the most important things about a leader. I think that the first couple of years you have to develop your confidence in your clinical area and get your boards through. I think the skill set that helped me the most was being open to possibility. I did not say no to things. I think you miss out on opportunities. I think being open to possibility is an important skill set.
Are women leaders necessary?	I think one of the most important things is the critical mass. We need more women in leadership roles because that will make this a continued process without it being a battle to keep us there. Gender balance in leadership brings a more diverse and innovative range of ideas.
Do women leaders need to be like men?	The women who are coming up I think are still having the same baggage that we did years ago: I am not good enough. This is the imposter thing that people talk about. I mean, we have that, we all have that big-time but women have it bigger than men. Most women are less competitive, because I think women are socialized more to work in groups, which is why they are better collaborators. I do not think male mentors would talk to anyone about their dress, even though I have seen women hurt themselves in a professional environment by the way they presented themselves. When a woman is offered a good position, she might say ‘Oh, I am not ready yet, I have to study, I have to do a number of things first to do an excellent job from day one and I do not yet know how to do it.’ A lot of men would say, ‘That sounds like a cool challenge and I’ll try it’.
Gender bias	I think that women need to be aware that there is still gender bias. It may not be overt, it may not be as wide spread as it was but there is still gender bias. And when we as women assume that there is none we are doing a disservice to other women. I think women need to be aware that there is still a bias out there and take that into account when they make decisions.
Women sought out natural mentors and sponsors to facilitate career development.	
Are mentors necessary?	Mentors provide clear visioning about what people want for themselves. And it has to be real. Not what they should do, but what they really want to do.
Find mentors for content development and career advancement	Someone pointed out to me that one person cannot be your be all to end all whether it’s in marriage or your children or in choosing mentors. And that what you should do if you are smart is find someone to help you, who can help you with something specific that you need. I did broaden out who I asked for advice about certain things. One mentor may not be broad enough to cover all of your interests. Any doctor, leader, or teacher can act as informal mentors if we only observe and learn from what they are doing.
Sponsorship	Part of it is that we are not encouraged, we as women are not encouraging other women to get involved. You know there were people that had to sort of hit me over the side of the head to say you are ready. Move, go for it. The problem is we have a human affinity to sponsor people who are like us. I really was not looking for a leadership role, and yet I was requested by my program director and peers in my class… he very much encouraged me and offered his ongoing services to talk me through if I came to some bumps in the road that were challenging. Women cannot sit back and wait to be recognized. Women need to share their drives and goals with the right people who can help them succeed. It is not enough to work really hard and wonder why you are not recognized for it.
Establish supportive connections	I do think the networking is important, because men do networking much better. Women interested in leadership, a chair or a Dean, need a network. We need to get together; we need to spread the word that there is support out there.
Women leaders intentionally planned their out of work life to support their leadership role.	
Work/Life issues	Adaptations and accommodations we make around childbearing and family are very important, but the best thing you can do for a woman in that process is to tell her that you really believe in her ambition. We need to keep supporting them in feeling good about being super capable.
An important work focus was to help others achieve excellence.	
Identify and reinforce leadership potential	Do not be afraid to surround yourself with people who are better than you are. Because if you provide the right environment for them, they will grow, and you will grow with them. They will bring you their best, and you will be learning from them. Consciously look for leadership talent that you see. And start it early. I think that means starting with medical students. I think that means residency. You look for that early on. Who has that kind of leadership potential and start actually putting the idea in their heads? So, I might meet with one of my senior residents and just say you know I have noticed that you really have a skill set for leading people through. Have you ever thought about becoming a chair some day? Nobody’s talked to them about it, they have never thought about it. Put that little bit of seed of interest in their head and see what happens. I think we also must allow people to find their own way, and how to do it. I have always been very supportive and taken advantage of programs like that but I did not define myself by those programs. The idea is to be a human being and citizen and not use gender as your defining feature.

#### Theme 1: women leaders made an intentional decision to pursue opportunities to influence emergency medicine

All of the leaders described a strong motivation to achieve a specific goal that led to a leadership role. *Sometimes it takes a mighty stimulus to propel us into leadership. In my case it was a sense of justice.*

Three leaders commented that they chose to lead *to have a voice in the policies that my organization is deciding because they will ultimately affect me*.

##### What strategic decisions and intentional risks did women leaders make?

All of the leaders described intentional choices they made before assuming leadership roles. Examples included training outside EM and understanding saying yes to something meant saying no to something else. *You cannot be risk adverse and be an effective leader*.

The earlier people can identify who they are and what they want out of life and a career, the clearer their vision will be. And once you have a clear vision, then the way you react to opportunities and invitations can be more deliberate. In the absence of a clear career vision, people can get overwhelmed and distracted by the opportunities available to them.

##### Are women leaders necessary?

Leaders agreed that empowering women and closing the gender leadership gap is essential for the future of emergency medicine. *We need to change our understanding of what men and women bring to the table, how we are programmed and wired differently*. *Together we are very compatible if we use each other’s strengths* versus *trying to have a masculine-only paradigm of leadership*.

All leaders agreed that more women need to be encouraged and offered leadership positions. *We need to make sure women get high profile opportunities where they are going to learn and get something back at the same time*.

##### Do women leaders need to be like men?

ᅟ

In the 80’s, books came out about how women need to be like men and now we have swung full pendulum to ‘women need to be women’. The answer is somewhere in between…women need to maintain their identities as women but still work in a field predominantly led by men. I think the first thing that women need to do is know who they are; know themselves.

I found that I had to take on masculine traits to be effective or at least to feel like it. … younger women do not see role models that they can relate to. For many of the first women to be in leadership positions, they usually must take on the attributes of the majority, who are men.

Subjects counseled younger women to deliberately adapt their presentation to appear more assertive: *Lower your voice and speak in short sentences. Wear glasses and your white coat to appear authoritative. The whole world has relaxed a bit, but overall when business is done people expect business attire and I think as ER docs we do not appreciate that. We live in the world of scrubs*.

##### Do women leaders face gender bias?

Six leaders acknowledged the existence of a covert bias because people hire people they are comfortable with: *men tend to hire men into open leadership roles*. ...*it*’*s a cumulative thing. So as a medical student you do not see that much unconscious bias*. *Some might even argue, maybe there*’*s none. But as you go up the ladder, things begin to change. There are fewer women, fewer role models and pockets of unconscious bias that are significant. There are people doubting your ability in an unconscious biased kind of way*... *they are valuing you less because you are a woman*.

Four subjects told stories of overt bias, illustrating how both men and women acted from assumptions based on traditional women’s roles such as prioritizing home over work. S*tories of the bad things that have happened to your sisters need to be shared more explicitly*. *At the same time*, *we need to share stories of how we overcame difficulties*.

##### Do clinical skills matter?

Mastery of the EM field was essential before becoming a leader: *I made sure I was always good at patient care*. All the leaders were involved in major EM initiatives including establishing departments or training programs, serving as founding or first female academic chair, or implementing system-wide initiatives.

My role model cared enormously about patients; the patient came first and everything else came second.

#### Theme 2: female leaders sought out natural mentors and sponsors to facilitate career development

You must find institutions that are thoughtful about women and value the role that gender diversity brings to the work place and are willing to champion putting women into some of these roles. Obviously, women leaders can do that as well and we need to be very thoughtful about supporting each other through these searches. I cannot tell you how many women I told “Look! Go apply for this job and put me down as a reference.”

##### Are mentors necessary?

All the leaders met mentors naturally while pursuing a passion. *Mentoring may not involve formally establishing a relationship*. A primary benefit of mentor guidance was to understand institutional culture and “unwritten” rules (e.g., academic promotion process), understanding how to acquire and manage institutional budgets or manage human resources (HR). *I needed a mentor when I first took on managerial roles*. For three subjects, this involved some sort of HR crisis (e.g., firing someone), or in two cases the development of a new program. Mentoring was sought out when a trusted confidant was needed (*When I was too stressed out*) or for information to fill gaps in knowledge and experience.

Mentoring comes from someone who has experience about something that you do not, where you can learn from them and they can guide you.

##### What kinds of mentors do women leaders need?

*Think about your mentors in two ways*: *content mentors and career mentors*. *They will help you deal with goal setting, planning*, *and making a path to where you want to be*. All the interviewees endorsed career development that results from working with a senior leader but did not support formal mentoring assignment programs: one interviewee called it “episodic mentoring.”

##### Do women leaders need sponsors?

All the leaders asserted that there are not enough sponsors to champion opportunities for women. Sponsors were always highly placed individuals who advocated for the woman’s advancement. Four interviewees specifically contrasted “mentoring” and “sponsorship.” *We need to understand the role of sponsorship*. *It is the senior person looking down and seeing potential in somebody and pulling them along*. *The senior person should have enough seniority and success themselves so that when the company or the board wants to attack a women leader*, *they can speak out on her behalf and be willing to stand up and challenge*.

Sponsorship qualities included *putting me in a position*, *making the connections*, *giving me the roles*, *transferring the knowledge and positioning me in a way to use my talents*. *I think there needs to be more women thinking of each other for positions of leadership and awards*. *We need to make sure their names come up and we endorse women*’*s skill sets*.

##### What supportive connections do women leaders need?

Four interviews highlighted the role of connections in facilitating a difficult time in their lives. *She probably does not remember the conversation, but she saved me that night*. *They joke it is lonely at the top*, *but it is really lonely when you have to present something publicly in a neutral way but privately you feel very different*. Having supportive connections with other women leaders was valued for difficult personal situations or career crossroad decisions.

Leaders emphasized the need to be known to gain professional achievement. All the leaders described situations where they were introduced to people who enhanced their understanding of the profession. Half of the subjects credited their success to national organizational involvement and the resulting networks they developed, affiliations with people who have doors to open and to finding collaborators who are like-minded in their ambition and goals. *I presented a paper at a professional meeting and was thinking that nobody noticed*, *but an academic chair told me*, ‘*That*’*s an incredibly good paper*. *I*’*m going to vote for you for best paper of the year*’. *I got the award*, *but it was more important to have someone like that tell me she heard my presentation*, *was impressed and knew who I was.*

Five interviews discussed the contribution of Executive Leadership in Academic Medicine (ELAM) Program for Women [20].

#### Theme 3: leaders intentionally planned their out of work life to support their work

I would never suggest that a woman not give the time and attention needed to her home and to her family. Women are still doing most of their housework and most of their child care. I really caution women not to use home and family obligations as an excuse not to take on the challenges of leadership. Yes, it is challenging but I think when women hide behind domestic duties, they are not acknowledging the real challenge of leadership.

##### What work/life issues do women leaders face?

*Women must work at least 120*% *more than men just to stay even*. All subjects acknowledged EM leadership is demanding and influences all aspects of life. Leaders described aspects of their personal lives including raising children and partners who also had demanding careers. *Women must realize that they are not going to have a 50*/*50 split at home*. *Maybe in the future but it is not now.*

Four leaders communicated two strategies: intentional conversations with their spouses/partners to develop a purposeful approach for family management and hiring help. *I have one of the strongest marriages that I know*, *but it*’*s not an accident*. *We have worked hard for it*. Some hired family to help: *You realize that your time is valuable and that you must hire out services to somebody who might even do it better than you*; especially to bridge challenging seasons like raising children. *When your kids start getting into elementary school*, *they want you*. *When they were little*, *they did not care about who took care of them*, *but once they are in school*, *they did not want their grandmother or nanny*, *they want mom*. *So*, *you must be very creative once they go into school*.

#### Theme 4: an important focus for their work was to help others achieve excellence

##### How do women leaders identify and reinforce potential?

ᅟ

I particularly seek out women and say, ‘I think you are ready to run for the board.’ I am so grateful for two people having done that for me. I would never have run for that first role if they had not pushed me.

All the leaders prioritized *growing people* as an essential part of their job. *Every chair puts their own stamp on a place*. *My stamp is developing people*. *I had the privilege of being able to help identify career pathways and advanced training, so their careers were enriching*, *fulfilling and effective*. *I think part of succession planning is recruiting in a very thoughtful way*.

When I became chair, I was no longer interested in my own career; I was interested in the careers of others. My success was only going to happen if other people were successful.

I can still remember one person saying to me that it was my job to learn my boss’s job. It was also my job to teach my job to the person who would one day take my job.

##### Are women unconsciously biased toward women leaders?

One leader described a situation where another female leader undermined her efforts by spreading criticisms behind her back. *I was getting hints that this was going on*. *Other leaders were saying* “*You might need to get better control over your team*.” *She was more about preserving old relationships over allowing me to make changes toward improvement.*

Women who have scratched and clawed their way to the top on their own, and now just feel like “the rest of you are just wimps” because you did not do it, are doing us a disservice. What we need to do is bring each other along. We have a harder role than the men do to get there.

##### What did leaders do to encourage more women to lead?

ᅟ

You mature as a leader through incremental leadership experiences, starting with little things and then grow through a career...encourage female faculty to seek leadership roles to the committee level, task force level or clerkship level and more senior faculty need to foster and encourage it. They need to get into leadership roles outside of the department to expand perspectives a bit.

We sponsored institutional, permanent funding for programs that brought women together. Women coming up are still having the same baggage, especially the imposter thing. We all have that big-time but women have it bigger than men.

##### What changes need to be made to increase women leaders?

ᅟ

It’s a real struggle as a leader to keep it in balance. Maybe the question is not: “How do we get women to want to take on something that’s inherently counter healthy?” but instead: “How can we rewrite the roles so that a sensible person would want to do it?” … Maybe the answer is we need to change the role of a leader to something that’s attractive to smart and sensible people.

We need the critical mass. We need more women in leadership roles because that will make this a continued process without being a battle to keep us there. We need to educate current women leaders to learn how to bring those behind them up in a planned and deliberate way. That removes some of that unconscious bias because once people look over and see a woman there it reminds them, we must help each other.

## Discussion

Our study provides insights from female leaders in EM, supporting the value of women pursuing leadership. There is an acknowledged need to diversify leadership and improve gender-specific needs to develop women leaders in medicine [21, 22]. Female leaders describe a “leaky pipeline” created by poor retention of women due to multiple factors such as lack of support for work-life integration and compensation inequities [13]. Women report leaving male-dominated fields because of perceived workplace hostility, isolation, and lack of role models [23]. Our subjects described their experience as *not old style*, *overt sex discrimination*, but an organizational culture that does not understand how covert bias exists against women [24]. *We should be asking what it is that women bring to the table*, *how does that diversity in a group make us stronger*, *get men to understand it and not try to shoot it down.* Defined directives regarding department culture, including attention to gender bias and transparent and equal access to career building opportunities, can help speed the changes needed to ensure a more effective representation of women leaders in EM [6, 24].

Women leaders need to watch and sponsor emerging leaders in all learner groups, reflecting their potential, and encouraging them to prepare for leadership roles. Leadership in academic medicine is changing with reorientation of a largely autocratic, vertically oriented hierarchy into a more democratic, consensus-driven, and horizontally organized management structure [25]. This fundamental restructuring complements the values of the millennial generation who prioritize work-life balance, early and frequent promotion with rationales for meaningful work commitments. We need to “let go of imagining the career pipeline as a sequence of age-dependent steps in favor of milestones of skill and talent development decoupled from age or educational stage” (p. 397) [26]. This might involve emphasis on “informal teaching and learning experiences with a high level of variability and individualization” (p. 11) in contrast to the more structured process typically found in clinical education [19]. Leaders should employ relatively new skills like coaching or sponsorship to improve prospects for women leaders [27].

Data suggests that despite performing at the same level or higher, women lack confidence relative to men [28]. Women carry a general sense of being not “worthy” or qualified to assume higher leadership positions and these feelings often go unrecognized or unchallenged by supervisors and academic leaders [29]. Most interviewees described wrestling with the “imposter syndrome.” Several expressed their surprise that *someone thought they were qualified to advance to leadership*, and yet it was that conversation that opened doors to opportunities and boosted their sense of confidence.

Women leaders may be most challenged by bad behavior from each other. Women report experiences like being ignored, interrupted, mocked, or treated disrespectfully, mostly from other women and that while women admire female leaders they would rather work for a man [30, 31]. Female leaders may be viewed as too independent, violating feminine norms of “communal” behavior by being dominant and assertive, and these perspectives may result in an unconscious bias toward career advancement for women [32, 33]. Sheppard and Aquino hypothesized that this concern is exaggerated, however, with roots in gender stereotypes blaming women’s alleged deficiencies instead of a biased organizational culture [31]. Women need to intentionally pull each other into leadership, and sponsor programs to combat implicit bias inherent in the workplace [33]. The topic warrants more research.

EM needs more role models of work/life integration to retain women in leadership roles. Women bring bigger societal burdens to the workplace which challenges their ability to assume leadership roles [23, 34]. Women considering leadership may “feel caught in the sandwich generation, caring for children and for aging parents” ([22, 35] p. S8). While individuals reported some accommodations offered to them during seasons of childbirth or family crises, few described deliberate efforts to broadly support women [36]. The lack of institutionalization of work/life integration programs perpetuates the stereotype that leaders must neglect personal balance in order to be leaders. Intervention programs teaching women skills in career planning, negotiating, and leadership facilitate informal mentoring and increase self-efficacy toward leadership aspiration [20, 34, 37]. Participants commented most frequently that sessions encouraged them to develop a broader network of connections [34]. Program models such as the University of Pennsylvania project to improve workplace culture to better support women faculty or the University of California Davis Women in Medicine should be promoted [38, 39].

Leaders need to be intentional about linking “natural” mentors and stretching experiences to potential leaders. Emerging leaders have little access to senior management, and mid-level mentors (i.e., program directors) were often the first entre leaders had to enhanced opportunities. Mentorship becomes more complex as women leaders may model more traditional leadership styles embraced by older generations which may not meet the needs of junior faculty [25]. Mentoring includes encouraging leadership development, facilitating stretch assignments and promotions, and influencing skill development [40, 41].

### Limitations

This was a qualitative study limited by the subjective perspective of those interviewed. All but two subjects reflect one generation (“baby boomers” over the age of 45) and women in younger generations may have varying interests and approaches to their work. Further exploration of the impact of dual career matching and similar programs impact leadership choices should also be pursued. Future research should include the changing role of women in EM within the context of the national health care priorities. These dramatic shifts are growing the role of the EM leader beyond clinical care and could become barriers to women accepting leadership roles.

## Conclusion

Studies show that a critical mass of female leaders (at least 30%) boosts organizations by improving innovation and creating a caring workplace culture [3]. Female chairs typically lead departments with a higher proportion of female faculty [42], which also supports the need for more women in leadership positions. The results of our study can be used to encourage emerging leaders by highlighting the path taken by senior EM leaders.

## Abbreviations

AAWEP: American Association of Women Emergency Physicians
ACEP: American College of Emergency Physicians
ELAM: Executive Leadership in Academic Medicine
EM: Emergency medicine
ER: Emergency room
ICM: Intelligent career model
